# Dengue, chikungunya and zika virus coinfection: results of the national surveillance during the zika epidemic in Colombia

**DOI:** 10.1017/S095026881800359X

**Published:** 2019-01-30

**Authors:** Marcela Mercado-Reyes, Jorge Acosta-Reyes, Edgar Navarro-Lechuga, Sherill Corchuelo, Angélica Rico, Edgar Parra, Natalia Tolosa, Lissethe Pardo, Maritza González, Jorge Martìn-Rodriguez-Hernández, Luz Karime-Osorio, Martha Ospina-Martinez, Helena Rodriguez-Perea, Gaspar Del Rio-Pertuz, Diego Viasus

**Affiliations:** 1 Department of Research in Public Health, National Institute of Health, Bogota D.C., Colombia; 2 Department of Public Health, Health Sciences Division, Universidad del Norte, Barranquilla, Colombia; 3 Department of Network in Public Health, National Institute of Health, Bogota D.C., Colombia; 4 Department of Public Health Surveillance, National Institute of Health, Bogota D.C., Colombia; 5 Institute of Public Health, Javeriana University, Bogotá D.C., Colombia; 6 General Director, National Institute of Health, Bogota D.C., Colombia; 7 Department of Medicine, Health Sciences Division, Universidad del Norte and Hospital Universidad del Norte, Barranquilla, Colombia

**Keywords:** Coinfection, chikungunya, dengue, mortality, zika

## Abstract

Our objective was to determine the frequency of zika (ZIKV), chikungunya (CHIKV) and dengue (DENV) virus coinfection and describe the mortality cases that occurred during the epidemiologic surveillance of the ZIKV epidemic in Colombia. We analysed all cases of suspected ZIKV infection that were reported to the National Institute of Health (October 2015–December 2016). DENV, CHIKV and ZIKV RNA were detected in serum or tissue samples using polymerase chain reaction assay. Medical records of the fatal cases were reviewed. We identified that 23 871 samples were processed. The frequency of viral agents was 439 (1.84%) for DENV, 257 (1.07%) for CHIKV and 10118 (42.38%) for ZIKV. Thirty-four (0.14%) cases of coinfection were identified. The CHIKV–ZIKV coinfection was present in 28 cases (82.3%), DENV–CHIKV in three (8.8%) and DENV–ZIKV in three (8.8%). Seven (20.6%) coinfection cases were fatal (two DENV–CHIKV cases and five CHIKV–ZIKV cases). Two cases were foetal deaths and the others were related to neurological syndrome and sepsis. In conclusion, the frequency of arbovirus coinfection during epidemic of ZIKV was low, and CHIKV–ZIKV coinfection was the most common. Mortality was high among coinfection patients. The role of each virus in the mortality cases of coinfection warrants further studies.

## Introduction

In recent years, the zika (ZIKV), chikungunya (CHIKV) and dengue (DENV) viruses have produced a large number of epidemics worldwide, which has caused significant morbidity and mortality [1, 2]. DENV infection is frequently a self-limited disease. It is caused by one of the four single-stranded, positive-sense RNA viruses (DENV type 1 through type 4). Every year, 50–100 million cases of DENV infection are reported and 250 000 to 550 000 fatal cases are identified in more than 100 countries. The incidence of DENV infection has increased more than 20-fold in the last 50 years [3, 4]. In Colombia, the average incidence of DENV infection in endemic years is the 347.2 cases/100 000 inhabitants. Moreover, CHIKV was first isolated after a 1952–1953 epidemic in Tanzania. Outbreaks of CHIKV were subsequently identified in Asia and recently in one region of the Americas. Millions of cases of CHIKV infection have been reported including severe cases and deaths [5, 6]. Colombia reported the first epidemic of CHIKV in 2014–2015, with an incidence of 1359 cases/100 000 inhabitants. In the next years, the average incidence was 72.5 cases/100 000 inhabitants. Furthermore, ZIKV was isolated for the first time in 1947 and the first human infections were described in 1964 [7, 8]. In 2007, there was an outbreak on several islands in the State of Yap, Federated States of Micronesia, which resulted in an infection that compromised more than 70% of the population [9]. Subsequent outbreaks occurred on other Pacific islands. The first transmission of the virus in America was reported in 2015 in Brazil [10]. Colombia reported the first autochthone cases in 2015, with an incidence of 377.7 cases/100 000 inhabitants. ZIKV infection has been associated with the development of several neurological diseases including microcephaly and Guillain–Barre syndrome [11–13].

The transmission of these arboviruses is produced by the same vectors and simultaneous circulation of them occurs in many regions of the world. In addition, cases of concurrent infections have been previously reported [14–17], and some of these cases have been associated with mortality [18]. In a recent study in Nicaragua, ZIKV, CHIKV and DENV infections resulted in similar clinical presentations and coinfections were relatively common; among 263 patients, 192 patients tested positive for a single virus (monoinfections) and 71 (27%) patients tested positive for two or all three virus coinfections [14]. Therefore, physicians should consider coinfection when selecting microbiological testing in patients with acute febrile disease in regions were simultaneous circulation of the viruses occur. In areas without molecular diagnostic capabilities, sensitive and rapid tests are required.

The objective of the current study was to determine the frequency of coinfection in samples processed by the Arbovirus Laboratory of the Colombian National Institute of Health and to describe the mortality cases associated with coinfection that occurred during epidemiologic surveillance of the zika epidemic in Colombia.

## Methods

### Study design

According to the procedures established by the Colombian National Institute of Health, all patients with suspected infection of DENV [19], CHIKV [20] and ZIKV [21] have to be reported to the National Surveillance System in Public Health (SIVIGILA). The cases are then confirmed by the National Institute of Health through laboratory tests or histopathological findings and clinical features. During the ZIKV epidemic in Colombia, the ‘suspect’ cases of ZIKV infection were required to show the presence of a rash and an elevation of axillary body temperature greater than 37.2 °C. In addition, cases were required to show one or more of the following symptoms that are not explained by other medical conditions: non-purulent conjunctivitis or conjunctival hyperaemia, arthralgia or myalgias and headache or general malaise. Furthermore, the infected individual had to be living in a place less than 2200 m above sea level or in a country with confirmed circulation of ZIKV. The guidelines of the Ministry of Health and Social Protection stipulate that serum samples must be sent to the National Institute of Health for laboratory confirmation of ZIKV. In addition, post-mortem biopsies are required for fatal cases related to suspected infection of ZIKV [21]. The samples of the different organs should be preserved and forwarded using buffered formalin at 10%. Because the zika epidemic is a health emergency for public health purposes and according with Colombian regulations, an autopsy should be performed even when there is no consent of the relatives.

This cross-sectional study included all of the cases of suspected ZIKV infection that were notified to SIVIGILA during the ZIKV epidemic in Colombia (October 2015–December 2016). Initially, DENV, CHIKV and ZIKV RNA were detected in serum samples or tissue samples using the US Centres for Disease Control (CDC)-designed singleplex (October 2015–March 2016). Later, DENV, CHIKV and ZIKV RNA were detected by the multiplex (Trioplex) real-time reverse transcription-polymerase chain reaction (rRT-PCR) assay (April 2016–December 2016) [22]. The singleplex or multiplex rRT-PCR assays were performed in the Arbovirus Laboratory of the Colombian National Institute of Health. Moreover, we collected detailed clinical findings, including history, physical examination and haematological, biochemical, radiological and virologic results, from fatal cases that were laboratory confirmed for coinfection. In addition, histopathological examinations were performed when a tissue autopsy was available.

### Molecular analysis

The Trioplex rRT-PCR assay used the enzyme SuperScript III Platinum One-step. The oligonucleotides and probes were run in a 25 µl final reaction volume and they were DENV-F, DENV-R1, DENV-R2 and DENV-P for dengue; CHIKV-F, CHIKV-R and CHIKV-P for chikungunya and ZIKV-F, ZIKV-R and ZIKV-P for zika. The thermal profile consisted of a reverse transcription step at 50 °C for 30 min, activation of the enzyme at 95 °C for 5 min and 45 cycles of 95 °C for 15 s and 60 °C for 1 min for hybridisation and extension. For the singleplex rRT-PCR test we used the following primers and probes: ZIKV 1086, ZIKV 1162c, ZIKV 1107-FAM, CHIKV 6856, CHIKV 6981c and CHIKV 6919-FAM. The same enzyme and thermal conditions described above were used for the Trioplex. For DENV detection, we used conventional molecular techniques following the protocol described by Usme-Ciro *et al*. [23]. During the period of the study, the four serotypes for DENV were investigated.

### Histopathologic analysis

The tissue autopsies were processed for histopathological examinations in the Pathology Laboratory of Colombian National Institute of Health. Formalin-fixed tissues from fatal cases were processed, embedded in paraffin, and cut into 4 µm sections. The histopathological changes were examined on haematoxylin- and eosin-stained tissue sections (Thermo; Sigma) under light microscopy.

### Ethical aspects

Following the Colombian laws, because infection by DENV, CHIKV and ZIKV are events of interest in public health, and as National Institute of Health it is allowed to use the histological and clinical material for research purposes without informed consent, which includes anonymous disclosure of results.

## Results

**Fig. 1.**
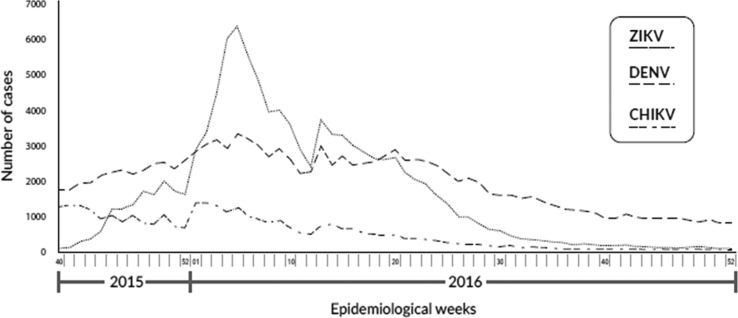
The distribution of patients with suspected infection by DENV, CHIKV and ZIKV reported to SIVIGILA during the ZIKV epidemic in Colombia (October 2015–December 2016). CHIKV, chikungunya virus; ZIKV, zika virus; DENV, dengue virus; SIVIGILA, National Surveillance System in Public Health.

The distribution of patients with suspected infection by DENV, CHIKV and ZIKV that was reported to SIVIGILA during the ZIKV epidemic in Colombia (October 2015–December 2016) is shown in Figure 1. During the study period, a decrease in the suspected cases of CHIKV was documented. However, there was an increase in suspected cases of DENV and ZIKV with a higher number of reported cases of ZIKV during the first weeks of 2016 (the epidemiological weeks from 1 to 10).

**Fig. 2.**
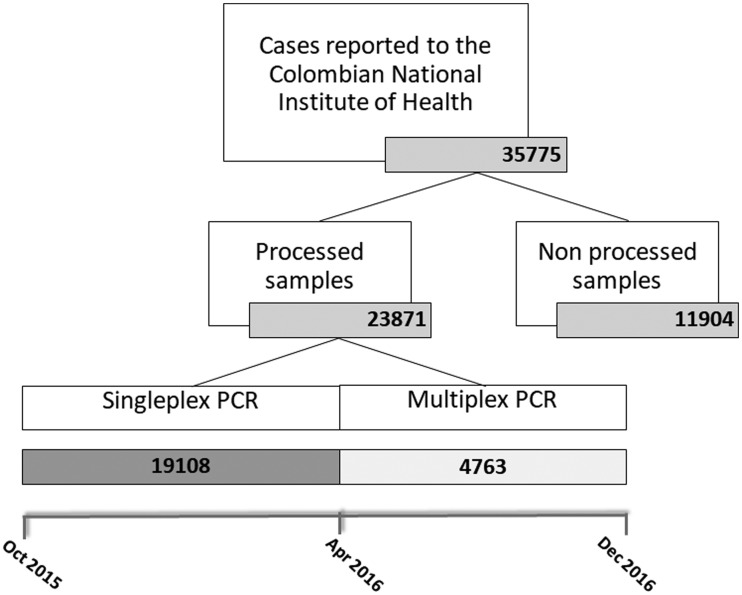
Number of cases reported and processed at National Institute of Health during the ZIKV epidemic in Colombia (October 2015–December 2016). PCR, polymerase chain reaction.

A total of 35 775 suspected cases of arbovirus infection were reported to the National Institute of Health during the ZIKV epidemic in Colombia, of which 11 904 were excluded for not complying to the procedures established by the Colombian National Institute of Health. Singleplex technique for ZIKV, CHIKV and DENV was used from October 2015 to March 2016 and was done to 19 108 cases. Trioplex technique was performed from April 2016 to December 2016 and was done to 4763 cases (Fig. 2). The frequency of the detected viral agents was 439 (1.84%) for DENV, 257 (1.07%) for CHIKV and 10 118 (42.38%) for ZIKV. The risk-population adjusted incidences in laboratory confirmed cases were 1.5/100 000 for DENV, 0.9/100 000 for CHIKV and 35.4/100 000 for ZIKV.

Thirty-four (0.14%) cases of coinfection were identified. The CHIKV–ZIKV coinfection was present in 28 cases (82.3%), DENV–CHIKV coinfection was present in three cases (8.8%) and DENV–ZIKV confection was present in three cases (8.8%). Regarding the DENV serotypes documented in coinfection cases, serotype 3 was present in all of the DENV–CHIKV coinfection cases. In the DENV–ZIKV coinfection cases, one had DENV serotype 2 and two had DENV serotype 3. The median age of all coinfection cases was 28 years (interquartile range 21–39) and 25 (73.5%) cases were female. Eight (23.5%) cases were less than 1 year old and four (11.7%) were 65 years old. Comorbidities were present in four (11.7%) cases, and 14 (41.2%) cases were pregnant women. Hospital admission was required in 26 (76.5%) cases.

**Fig. 3.**
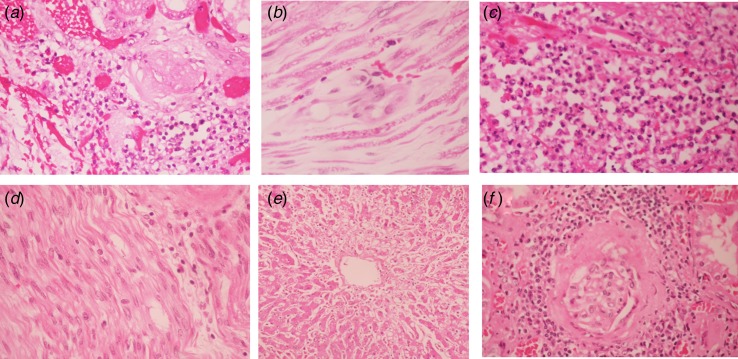
Histopathological findings among fatal adult cases with DENV, CHIKV and/or ZIKV coinfection during the ZIKV epidemic in Colombia (October 2015–December 2016). (a) Tubulointerstitial nephritis, (b) acute motor axonal polyneuropathy and (c) acute pneumonia found in case 2; (d) acute demyelinating polyneuropathy from case 4; (e) necrosis surrounding hepatic vein in case 3; and (f) tubulointerstitial nephritis in case 1. Magnification: e (20×) and a–e, F (40×). CHIKV, chikungunya virus; ZIKV, zika virus; DENV, dengue virus.

Among coinfection cases, seven (20.6%) were fatal (two DENV–CHIKV cases and five CHIKV–ZIKV cases). Five of the fatal cases were adults (Table 1) and two were foetal deaths (Table 2). Among the fatal adult cases, most of them had comorbidities and three were female. Three cases developed neurological symptoms and the other two had sepsis and multiorgan failure. The histopathologic findings included tubule-interstitial nephritis, acute tubular necrosis, acute motor axonal polyneuropathy, acute demyelinating polyneuropathy and pneumonia (Table 2). Regarding foetal deaths, one case had acrania and anencephaly that was diagnosed by ultrasound in week 15 of the pregnancy. The other foetal death was diagnosed in week 34 of the pregnancy by an absence of a foetal cardiac rate in the echocardiography. Some histopathological findings of adult fatal cases are shown in Figure 3.

**Table 1.** Clinical features and laboratory findings in fatal adult cases with virus coinfection during the ZIKV epidemic in Colombia

Virus coinfection	CHIKV–ZIKV	DENV–CHIKV	DENV–CHIKV	CHIKV–ZIKV	CHIKV–ZIKV
*Clinical features*					
Age (years)	47	71	54	78	28
Comorbidities	Hypertension Diabetes mellitus Obesity	Hypertension Smoker	Hypertension Smoker	Hypertension Diabetes mellitus Chronic kidney disease Chronic cardiac disease	No
Time from symptom onset to admission (days)	2	3	3	1	2
Fever	No	No	Yes	No	Yes
Arthralgia	No	No	Yes	No	Yes
Rash	No	No	No	No	No
Haemorrhagic manifestations	No	No	Yes	No	Yes
Oedema	No	No	No	No	No
Time from admission to mortality (days)	9	10	1	15	2
Complications	Neurological syndrome	Neurological syndrome	Hepatic failure Acute kidney injury Pneumonia	Neurological syndrome	Multiple organ failure
*Laboratory findings at admission*					
Haemoglobin (g/dl)	13	NA	13.8	14.8	14.6
Haematocrit (%)	41	NA	40.5	42.7	41
Total white cells (10/l)	10 100	NA	2400	4800	9500
Platelets (10/l)	320 000	NA	8000	103 000	306 000
Creatinine (mg/dl)	0.66	NA	1.91	0.89	0.6
Aspartate aminotransferase (U/l)	NA	NA	987	28.9	550
Alanine aminotransferase (U/l)	NA	NA	1478	23.1	150
*Histopathologic findings*	Tubule-interstitial nephritis, changes related to SIRS	Acute motor axonal polyneuropathy, CNS without alterations, coagulative non-confluent zonal liver necrosis pneumonia, tubule-interstitial nephritis	Coagulative non-confluent zonal liver necrosis, acute tubular necrosis, spleen with reactive plasmacytosis, pulmonary haemorrhage, changes related to SIRS	Acute demyelinating polyneuropathy, pneumonia, changes related to SIRS in liver and spleen	NA

NA, not available; CHIKV, chikungunya virus; ZIKV, zika virus; DENV, dengue virus; CNS, central nervous system; SIRS, systemic inflammatory response syndrome.

Normal ranges: Haemoglobin: 12–17 g/dl; haematocrit: 40–50%; platelets: 150–600 × 10^9^/l; total white cells: 4–12 × 10^9^/l; creatinine: 0.75–1.2 mg/dl; aspartate aminotransferase 10–34 U/l; alanine aminotransferase: 5–59 U/l.

**Table 2.** Ultrasonographical and pathologic findings from foetal death cases with virus coinfection during the ZIKV epidemic in Colombia

	Case 6	Case 7
Virus co-infection	CHIKV–ZIKV	CHIKV–ZIKV
Mother age (years)	22	22
Ultrasonographical findings	Intrauterine growth restriction, anencephaly	Overriding of skull bones, absence of heartbeats
Histopathologic findings	Trivascular umbilical cord without inflammatory process, thymus, kidney, spleen and intestinal wall with diffuse congestion, third trimester chorionic villi without inflammatory process, liver with extramedullary haematopoiesis	Autolysis changes that didn't allow a morphologic evaluation, lungs in the alveolar development stage, myocardium without collagen remodulation, kidney sample shows normal nephroblast development

CHIKV, chikungunya virus; ZIKV, zika virus.

## Discussion

In the current study, we evaluated the frequency of arbovirus coinfection and describe the mortality cases associated with coinfection that occurred during national surveillance of the zika epidemic in Colombia. The frequency of arbovirus (DENV, CHIKV and/or ZIKV) coinfection was low and CHIKV–ZIKV coinfection was the most common. Seven cases were fatal among the coinfection patients.

Simultaneous circulation of ZIKV, CHIKV and/or DENV has been documented in many regions of the world. Some studies report that DENV, CHIKV and/or ZIKV coinfection is not uncommon. In a study performed in Nicaragua, Waggoner *et al*. [14] documented that ZIKV, CHIKV and DENV had similar clinical presentations. In addition, coinfections were found in 71 (26.9%) of 263 patients that tested positive for one or more virus. Furthermore, mean viraemia was significantly lower in coinfections compared to monoinfections. CHIKV–DENV coinfection was the most common (60.5%). Moreover, Chia *et al*. [24] performed a study during a ZIKV outbreak in Singapore and described ZIKV–DENV coinfections. Of the total of 163 cases of ZIKV infection that were identified, five cases (3.5%) were positive DENV coinfection. Otherwise, the clinical features of coinfected patients did not significantly differ from reported ZIKV monoinfections and worse outcomes were not documented. In another study conducted in a city in the northeast region of Brazil during a febrile outbreak in 2015, the coinfection of DENV and ZIKV was detected in two of 77 total patients [16]. Moreover, coinfections of DENV–CHIKV also were documented during CHIKV epidemics [18, 25].

Consistent with previous reports about ZIKV outbreaks that occurred in different areas of the world and where there was simultaneous circulation of arbovirus, our results also showed that acute febrile illness can be caused by ZIKV, DENV and CHIKV in these regions. However, our coinfection rate was lower than that reported in previous studies. In this regard, the current study was based on a large number of samples from all Colombian regions that were sent to the National Institute of Health due to the national surveillance performed by the government. The samples were originated from ambulatory and hospitalised patients. Moreover, the frequency of arbovirus involved in coinfection cases was also different in the current study compared to that reported from other studies. Most coinfection cases of our study were caused by CHIKV–ZIKV. Conversely, in a study performed in Nicaragua, CHIKV–DENV coinfection was the most common [14].

In the current study, a description of the fatal coinfection cases that occurred during the ZIKV epidemic in Colombia was performed. Foetal mortality cases were associated with ZIKV–CHIKV coinfection. Similarly, a case of foetal death associated with coinfection by ZIKV–CHIKV in a Brazilian pregnant woman has been reported [26]. The foetus had no apparent anomalies. In this regard, ZIKV infection during pregnancy has been associated with the development of several foetal neurological diseases and potentially associated abnormalities of the eye, brain and placenta. Amniotic fluid and tissues from multiple foetal organs have been shown to test positive for ZIKV [27–29]. Moreover, in a recent study performed in Reunion Island, pregnant women infected by CHIKV had no observable effect on outcomes (caesarean deliveries, obstetric haemorrhaging, preterm births, stillbirths after 22 weeks, birthweight, congenital malformations and new-born admissions) [30]. However, some reported cases of foetal mortality have been directly linked to the maternal-foetal transmission of CHIKV [31]. In addition, some fatal adult cases that were documented in the current study were related to the development of neurological symptoms and infectious nosocomial infections, and these cases were related to ZIKV–CHIKV coinfection. In previous studies, both CHIKV and ZIKV have been related to the development of Guillain–Barre syndrome [12, 32, 33]. However, the role of each virus in the mortality or severity of the illness in patients with coinfection is not known. As a hypothesis, an increase in the immune response or susceptibility of the host for a more serious disease when coinfection occurs could explain our findings. However, future studies are needed to clarify these aspects.

Simultaneous circulation of the three arboviruses may confuse clinicians because ZIKV, CHIKV and DENV mono- and coinfections have similar clinical presentations [14, 25]. This could result in possibly omitting one differential diagnosis that could impact the prognosis in special populations (pregnant women, children less than 1 year old, the population over 60 years old and people with comorbidities). These data support the need of sensitive and rapid tests in areas where the three arboviruses are present due to the importance in differentiating between the viruses and because distinct management and follow-up is needed for each virus.

The strength of the current study is that it was based on a large number of samples from a national surveillance during a ZIKV epidemic in Colombia. The cases were from children and adults from geographically diverse regions across the country. In addition, we evaluated the clinical and histopathological features of the fatal patients. However, there are some limitations to our study. Because of the retrospective design of the study, we did not have complete medical records. Also, it is possible that other cases were not reported to the SIVIGILA. Moreover, immunohistochemical and serological studies were not performed.

In conclusion, the frequency of arbovirus (DENV, CHIKV and/or ZIKV) coinfection during the ZIKV epidemic in Colombia was low and CHIKV–ZIKV coinfection was the most common. Seven cases were fatal among the coinfection patients and were mainly foetal deaths and adult mortality cases were related to neurological complications and sepsis. Determination of the role of each virus in the mortality of patients with coinfection warrants further study. Importantly, our findings highlight the need to consider accurate diagnostic tests for patients with suspected febrile illness and as part of epidemiologic surveillance.
